# Searching for the Achilles Heel of FOXP3

**DOI:** 10.3389/fonc.2013.00294

**Published:** 2013-12-03

**Authors:** Teresa Lozano, Noelia Casares, Juan José Lasarte

**Affiliations:** ^1^Gene Therapy and Hepatology Area, Center for Applied Medical Research (CIMA), University of Navarra, Pamplona, Spain

**Keywords:** foxp3, FOXP3 interactome, Treg, drug discovery, immunosuppression

## Abstract

FOXP3 is a multifaceted transcription factor with a major role in the control of immune homeostasis mediated by T regulatory cells (Treg). The immunoregulatory function of FOXP3 may hinder the induction of immune responses against cancer and infectious agents, and thus, development of inhibitors of its functions might give new therapeutic opportunities for these diseases. But also, FOXP3 is an important tumor suppressor factor in some types of cancers, and therefore, understanding the structure and function of FOXP3 is crucial to gaining insights into the development of FOXP3-targeted therapeutic strategies. FOXP3 homodimerize and likely form supramolecular complexes which might include hundreds of proteins which constitute the FOXP3 interactome. Many of the functions of FOXP3 are clearly regulated by the interactions with these cofactors contributing importantly on the establishment of Treg-cell signature. We summarize here the structural/functional information on this FOXP3 complex, to identify potential opportunities for the development of new strategies to modulate FOXP3 activity.

## FOXP3, a Member of Fox Family, with Immunoregulatory Properties

Forkhead (FKH) proteins are a very large family of transcription factors implicated in a variety of cellular processes (1). They are characterized by the presence of a highly conserved 100-amino-acid FKH-binding domain (FKH). The crystal structure of the FKH domain bound to DNA has been solved and has been described as a “winged-helix” similar to the shape of a butterfly (2).

In the last years, more than 100 members, classified in 15 subfamilies of FOX proteins, have been identified (3). They are commonly associated with the regulation of development. Whereas the FKH domain defines this class of transcription factors, other portions of the Fox proteins, which encode for instance transactivation or trans-repression domains, are highly divergent and little is known about their interactions with the transcriptional machinery.

FOX gene mutations have been found to be responsible for diverse phenotypes, from craniopharyngeal development (FOXE1), hair development (Foxq1 and Foxn1) to speech and language development (FOXP2) and hearing (Foxi1) (4). In the last years, it has become clear that these winged-helix proteins have crucial roles in various aspects of immune regulation. Thus, the scurfy mutant mouse strain, which suffers from a fatal lymphoproliferative disease leading to early death within 3–4 weeks of age, presents a frame-shift mutation of the FKH box transcription factor Foxp3 responsible of this disease (5). This affection is characterized by splenomegaly, insulitis, hepatomegaly, lymphadenopathy, and massive lymphocytic infiltrates in the skin and liver, suggesting that mutation in FOXP3 leads to an autoimmune disease.

In humans, mutations in FOXP3 results in an autoimmune syndrome termed IPEX (immunodysregulation, polyendocrinopathy enteropathy, X-linked syndrome), an X-linked immunodeficiency syndrome characterized by insulin-dependent diabetes, thyroiditis, massive T-cell infiltration in multiple organs and chronic wasting (6–8).

Analysis of FOXP3 expression revealed that it was most highly expressed in lymphoid organs, such as the thymus and spleen, and more specifically by CD4+ T cells. The relevance of Foxp3 expression in CD4+ T cells was evidenced when it was found that adoptive transfer of wild-type CD4+CD8− lymphocytes could control the T cells of scurfy mice, thereby preventing the development of disease (9). This observation suggested that CD4+ T cells expressing the wild-type Foxp3 gene played a regulatory role being able to suppress the activity of other immune cells. This was confirmed when CD4+CD25+ T cells were identified as cells able of suppressing proliferation and cytokine production of TCR-stimulated conventional CD4+ T cells (10, 11). Foxp3 expression in CD4+ T cells was found to be sufficient to mark these cells as regulatory (Treg cells). Indeed, infection of CD4+CD25− T cells with a retrovirus expressing Foxp3, converted them to a regulatory phenotype, able to suppress proliferation of conventional CD4+ CD25− T cells (12) and protect mice from autoimmune gastritis. These studies demonstrated that CD4+CD25+ T regulatory cells with a major role in the suppression of autoreactive T cells, and preventing autoimmunity. The activity of Foxp3 was also confirmed in transgenic mice overexpressing Foxp3 (13), and curiously, it was found that also CD8+ T cells overexpressing Foxp3 displayed *in vitro* regulatory activity, showing that Foxp3 expression on non-CD4 T cells was also capable to re-program the cell phenotype.

## FOXP3 and Cancer

Treg cells inhibit activation of other T cells and are needed for protection against autoimmune diseases. However, immunoregulatory function of Treg may hinder the induction of immune responses against cancer and infectious agents (14–20). Indeed, Treg capable of suppressing the *in vitro* function of tumor-reactive T cells have been found in humans in many types of tumors (16–18, 20) and have been associated with a high death hazard and reduced survival (16–18). However, there are discrepancies in the prognostic studies relying on the presence of Treg in tumor infiltrates, and paradoxically, a high density of FOXP3+ T-cell infiltration was associated with improved overall survival in patients with colorectal, head and neck carcinoma, or patients with lymphoma (21–23). It has been postulated that Treg could in these cases modulate the tumor microenvironment and influence the biologic behavior of tumoral cells. A better understanding of the biologic role of FOXP3-positive Tregs in these tumors is needed.

Despite a clear role in Tregs, FOXP3 protein expression is not restricted to the lymphocyte lineage but is also present in normal and cancer cells of non-hematopoietic origin (24–28). The function of FOXP3 in cancer is somehow contradictory. Regarding the expression of FOXP3 in human cancer cells and in their normal homologs, two opposite situations have been found. It has been described that in pancreatic cancers or in melanoma, FOXP3 expression is restricted to the tumor cells (24, 25). In contrast, FOXP3 appears to be expressed in normal epithelial cells of human breast and prostate, but downregulated in the corresponding cancer cells (29, 30). These data suggest a dual role of FOXP3, one linked to immune escape and another to tumor suppression (31).

On the one hand, it has been shown that FOXP3 expression in melanoma cells (26) or in pancreatic carcinoma cells (25) renders the tumor cells suppressive with Treg-like activity to directly inhibit the proliferation of T cells and suggesting a possible mechanism of tumor resistance to immune system. However, on the other hand, several works have demonstrated that the expression of FOXP3, especially in breast cancer cells, is an X-linked cancer suppressor gene and an important regulator of the epidermal growth factor receptor (HER2/ErbB2) and SKP2 oncogenes (27, 30). The expression of Foxp3 has been evidenced in a significant number of cancer types, although its role in tumor progression remains to be elucidated (32).

## The Interactome of FOXP3

FOXP3 is essential for the specification and maintenance of Treg cells, and thus, it was considered as the “master regulator” of Treg cells although it was described that cells with many of the Treg-cell characteristics, can differentiate at least transiently, in the absence of FOXP3 (33, 34). The molecular basis of FOXP3 function has been poorly understood. As described above, genome-wide analyses of Foxp3 targets has revealed that FOXP3 induces both activation and repression of its target genes (33–37). The capacity of FOXP3 to bind DNA is critical for its functionality; however, it is clear that FOXP3-DNA interactions are assisted by FOXP3 cofactors and by multimerization. After a careful meta-analysis that combined gene-expression profiles, generated in several parallel experiments, Hill et al. identified 603 target genes (407 overexpressed and 196 underexpressed) that compose the canonical Treg-cell signature (38). Importantly, it was found that much of the Treg-cell signature was not ascribable to Foxp3 because it contained gene clusters that are co-regulated with, but not transactivated by FOXP3. Recently, Samstein et al. (39) examined chromatin accessibility of FOXP3-bound enhancers in Treg cells and Foxp3^-^ CD4^+^ T cells. They showed that FOXP3 was bound mainly to enhancers already accessible in precursor CD4^+^ FOXP3^-^ T cells, with only a small subset of exclusively Treg-restricted enhancers found in several genes important for Treg-cell function. Analysis of DNA sequences at FOXP3-binding sites identified a FKH motif only in a small subset of these DNA regions (39), suggesting the contribution of other cofactors on the establishment of Treg-cell signature.

Indeed, it has been described that FOXP3 homodimerize and likely form supramolecular complexes (40). Growing numbers of transcription factors that interact with FOXP3 are being identified and some have been implicated in the Treg-cell-specific gene-expression program (37, 40, 41). In a recent paper, a chromatographic analyses of nuclear lysates revealed that FOXP3 protein was present in large 400–2,000 kDa complexes (41). In this work, Rudra et al. identified 361 partners of FOXP3 by using a proteomic approach. They found that these partners were implicated in several biological processes such as DNA binding, transcription regulator activity, chromatin binding, regulation of transcription, chromosome organization, chromatin modification as well as RNA binding, processing, splicing, and metabolism suggesting a yet unexplored RNA-associated role of FOXP3. Recently, Fu et al. by using computational network inference, confirmed that FOXP3 expression was responsible of around 10% of the whole Treg signature and identified five transcription factors (Eos, IRF4, Satb1, Lef1, and GATA-1), which they named “the quintet” that interact with and act together with FOXP3 to elicit much of the characteristic Treg-cell transcriptional signature (37).

This observation is consistent with the fact that the majority of FOXP3-binding sites in the genome lack an identifiable FKH-binding motif in Treg cells and suggests that FOXP3 cofactors may facilitate binding of FOXP3 to a given site either through direct recruitment of FOXP3-containing complexes or through facilitating interactions with FOXP3-bound sites containing FKH motif via loop formation (35, 36, 39). It is clear that FOXP3 functions, and consequently the phenotype of the FOXP3-expressing cell may change depending on the partners forming the FOXP3 interactome, which might in addition, depend on the activation status of the cell.

A significant number of FOXP3-interacting proteins have been identified, including TIP60, HDAC7, HDAC9, Eos, Irf4, and Hif-1α (40, 42–48). The proteomic analysis of FOXP3-complexes conducted by Rudra et al., found that the majority of FOXP3 partners consisted of proteins that have been implicated in regulation of transcription including many sequence-specific transcription factors such as NFATc2, Runx1, Bcl11b, Foxp1, Foxp4, GATA-3, STAT3, Ikaros (Ikzf1), Aiolos (Ikzf3), Ets, and Cnot3. It has been postulated that the differentiated state of Treg cells is not determined by individual regulatory components, but instead by the collective activity of their transcriptional network (37, 41, 49). This hypothesis has important implications on the functionality of Tregs, and raises new possibilities for the design of new therapies where Treg cells or FOXP3 expression and function have pathological consequences. Indeed, those strategies able to inhibit a particular interaction with FOXP3, or able to modify the FOXP3 interactome might have important consequences on the whole transcriptome signature of the FOXP3-expressing cell and consequently, on its activity. Thus, a structure/function analysis of FOXP3 might help on the understanding of the rules governing the formation of such interactome.

## The Structure/Function Analysis of FOXP3

The *FOXP3* gene is located on the X chromosome and contains 11 coding exons (exons 1–11) and 3 non-coding exons (5). FOXP3 has various distinguishable functional domains: (i) an N-terminal domain (from a.a. 1 to 193, with two proline-rich regions) responsible for transcriptional repression, (ii) a zinc finger (a.a. 200–223) and a leucine-zipper (LZ)-like motif (a.a. 240–261) (ZL domain) located in the center of the protein, which facilitates the formation of FOXP3 homo-dimers or tetramers, and (iii) the highly conserved carboxy terminal FKH domain (FKH; from a.a. 338 to 421) responsible for the DNA binding. The analysis of FOXP3 variants containing mutations found in IPEX patients have offered some evidences about the function of these domains. Mutations have been found throughout FOXP3 indicating that these regions of the protein are important for proper function (50, 51). Identification of the functional consequences of these individual mutations is critical to gain insights into possible treatments for Foxp3-related pathologies.

### The amino terminal region of FOXP3

The N-terminal of FOXP3 has been involved in the interaction with several transcription factors, which are related to the suppressive transcriptional activity of FOXP3. Lopes et al. evaluated the location of point mutations identified in a large cohort of patients with the IPEX syndrome, and found them to cluster primarily in the FKH domain and the leucine zipper, but also in the N-terminal portion of the protein (51). They identified a functional domain in this N-terminal region of FOXP3 which is required for FOXP3-mediated repression of transcription. They mapped the functional repressor domain between aminoacids 67 and 132.

One of the key features of Treg cells is to maintain the anergic status in response to stimulation. In this regard, Lee et al. found that the N-terminus of FOXP3 specifically interacts with and alters subnuclear localization of phosphorylated c-Jun (52), a member of the AP-1 transcription factors which participates in the control of cell proliferation, differentiation, cell death, and cell transformation in response to stimuli (53). This interaction inhibits the promoter DNA-binding activity of c-Jun and induces T-cell anergy.

As mentioned above, FOXP3 expression is not restricted to the lymphocyte lineage but it is also present in cancer cells, especially in breast cancer cells, where it has been demonstrated to be a cancer suppressor gene and an important regulator of the HER2/ErbB2 and SKP2 oncogenes (27, 30). It has been described that this tumor suppressor activity is located in the N-terminal region of Foxp3, since a C-truncated versions of the protein do not retain this property (54). The authors described that this activity was independent of NFAT-FOXP3 interaction which is located in the FKH region of FOXP3 (55). It is tempting to postulate that the tumor suppressor activity of Foxp3 is mediated by its capacity to alter the activation of AP-1 through its interaction with c-Jun.

The suppression of CD4+ T cells proliferation is also maintained through the interaction of Foxp3 N-terminal domain with Eos, a zinc-finger transcription factor of the Ikaros family (45). Foxp3, Eos, and the C-terminal binding protein-1 (CtBP1), form an inhibitory complex that suppresses the expression of genes. Disruption of the interaction of Foxp3 and Eos leads to the failure of Treg to inhibit proliferation of T effector cells. The proline rich N-terminal region of FOXP3 seems to be the responsible for this interaction. However, this region alone is not sufficient to confer Treg cells suppressive function, and NFAT-FOXP3 interaction through the FKH domain is also required (46, 52, 55). In a recent paper, a new human-specific FOXP3-interacting protein which couples FOXP3 to a chromatin-remodeling complex has been identified to contact with this region (aa 106–198) (56). The authors show that FIK protein (FOXP3-interacting KRAB domain containing protein), acts as a bridging molecule to link FOXP3 with the chromatin-remodeling scaffold protein KAP1 (TIF-1β/TRIM28) and that disruption of this complex restores the expression of genes inhibited by FOXP3, and the suppressor activity of Tregs. Interestingly, expression of genes that were shown to be positively regulated by FOXP3 (such as CTLA4 or CD25) was not affected, suggesting that they were regulated by a different mechanism.

In humans, there are two FOXP3 isoforms expressed in Treg cells that lacks exon 2 (57), located in the N-terminal region of FOXP3. It has been described that exon 2 is implicated in the interaction with RORα and RORγt, transcriptional factors that activate the expression of IL-17 and IL-22, which are related with Th17 differentiation. RORγt binds to IL-17A promoter and the interaction of Foxp3 with RORγt through exon 2 region of Foxp3 is able to suppress of RORγt-mediated IL-17A promoter activation. These results highlight the importance of Foxp3 N-terminal domain in balancing the differentiation between Treg cells and Th17 (58, 59).

The N-terminal of FOXP3 is also reported to interact with c-Rel (60, 61), a member of the NF-κB family that is responsible for the up-regulation of a variety of pro-inflammatory cytokines such as IL-6, IL-12, IL-15, IFN-γ, or IL-2 (62, 63). Deletion of exon 1 was sufficient to almost entirely abrogate c-Rel binding, although other regions of FOXP3, such as exon 6 or exon 8 also abolished binding, suggesting that dimerization of Foxp3 or the interaction with other proteins is required. The interaction between Foxp3 and c-Rel may also contribute to the nuclear translocation of FOXP3. Indeed, it has been shown that FKH-deleted FOXP3, which is mainly located at cytoplasm, was predominantly found in the nucleus in the presence of ectopically expressed c-Rel (60, 61).

In addition to the interaction with a number of transcriptional factors, FOXP3 also interacts with enzymes that regulate the activity of FOXP3 at post-translational level [reviewed in (64)]. Among these enzymes, histone acetyltransferase (HAT) TIP60 and histone deacetylase HDAC7 interact with the N-terminal of FOXP3 and increase its suppressive transcriptional activity (43). TIP60 promotes the acetylation of FOXP3, which enhances the suppressive transcriptional activity of FOXP3, but TIP60 may also play a role in regulating FOXP3 activity independent of its HAT activity. The N-terminal 106–190 aa of FOXP3 are required for TIP60-FOXP3, HDAC7-FOXP3 association, as well as for the transcriptional repression of FOXP3 via its FKH domain (43). Binding of TIP60 with FOXP3 may regulate the oligomerization status of FOXP3 and therefore its DNA binding. Moreover, TIP60 may also recruit additional factors required for transcriptional repression/activation. A clear understanding of the involvement of HDACs in the FOXP3 complex assembly will lead to insights to develop new strategies to modulate Treg functions for human disease (64).

Several of these interactions have been linked to a particular Treg-cell transcriptional profile. This is the case for IRF4 or STAT3, which can affect, alone or in combination with FOXP3, some segments of the Treg-cell signature (47, 65). Their presence should modulate the range of Treg effector functions. In this way, it has been reported that a modification of the N-terminus of Foxp3, results in diametrically opposite effects in the severity of different autoimmune diseases. Mice expressing the GFP-Foxp3 fusion protein modified Foxp3 molecular interactions, blocking HIF-1α but increasing IRF4 interactions and resulted in a divergent susceptibility to autoimmune disease: protection against antibody-mediated arthritis, but greater susceptibility to diabetes (66). Thus, specific subfunctions of Treg cells and the immune diseases they regulate can be influenced by Foxp3’s molecular interactions with particular partners, which result in different patterns of immunoregulation.

### Zinc finger and leucine zipper

Dimerization of FOXP3 is required for its function as a transcriptional regulator and the LZ of FOXP3 is necessary and sufficient for this association (40, 51, 64, 67). In addition, the LZ of FOXP3 is implicated in the hetero-association with FOXP1 (64, 68). The disease-associated mutations of the LZ domain disrupting FOXP3 dimerization can substantially reduce the binding of FOXP3 to promoter regions *in vivo* (40, 51, 64, 67). These findings indicate that the FOXP3 protein ensemble, as well as its DNA-binding properties, could be modulated through oligomerization of the LZ region. Song et al. have characterized the structure of the FOXP3-Zinc finger and LZ (FOXP3-ZL) domain and identified two lysine residues in the LZ region (K250 and K252) as key residues for the conformational change and stability of the FOXP3 dimer. Acetylation of these K residues would neutralize the positive charges at these sites through which the FOXP3 dimer stability is modulated and result in changes in promoter occupancy, histone acetylation patterns, IL-2 gene-expression levels, and Treg suppression activity.

Moreover, the LZ is also important for the interaction between histone H1.5 and FOXP3, which cooperatively repress IL-2 transcription in human T cells. This interaction alters histone H1.5 binding to target genes (i.e., IL-2 or CTLA4) to modulate their expression and to program Treg function (69). Also, FOXP1-FOXP3 hetero-associations through the LZ domain may compete with FOXP3 homo-dimerization and the dynamic balance of discrete forms of FOXP3-complexes may directly affect its repressor activity (69).

FOXP3 interacts physically with the transcription factor AML1 (acute myeloid leukemia 1)/Runx1 (Runt-related transcription factor 1) (44), which is crucially required for normal hematopoiesis including thymic T-cell development. Ono et al. showed that this interaction suppresses IL-2 and IFN-γ production, upregulates Treg-associated molecules, controls anergy of the cell and exerts suppressive activity. They also found that the AML1-interacting domain of the Foxp3 protein was located between the FKH domain and the LZ motif (amino acids 278–336). Three amino acids located immediately N-terminal to the FKH domain are important for this inhibition, and their mutation (D329V, Y330H, and K332L) impaired Treg suppressive function. Thus, the interaction between AML1 and FOXP3 may be another potential therapeutic target for controlling Treg activity.

### FKH domain

As a member of Fox family, FOXP3 contains a FKH domain responsible for DNA binding and consequently for its activator/repressor functions. More than half of patients with IPEX syndrome have missense mutations in exons encoding the FKH domain (70–74). Mutations in the FKH domain of patients with IPEX have been shown to affect DNA binding (8, 51, 75). FOXP3-binding to DNA requires multimerization and it has been shown that FKH domain of FOXP3 forms a domain-swapped dimer, simultaneously engaging and bringing into close proximity two distant FOXP3-binding sites in DNA (55). Thus, it seems that both the DNA-binding FKH domain and an intact LZ domain of FOXP3 are needed for homo-multimerization of FOXP3, DNA binding, and consequently for FOXP3-mediated suppressor functions (55, 76).

**Figure 1:**
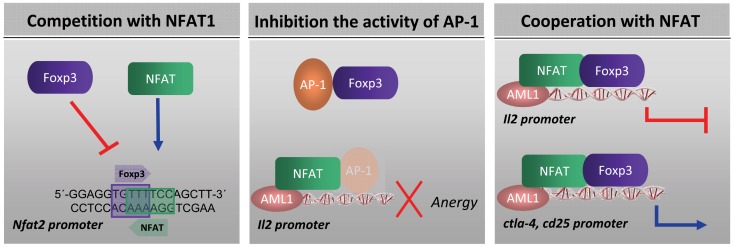
**Mechanism by which FOXP3 influences the expression of NFAT-dependent genes: (i) by competing with NFAT for binding to DNA (77–79) (ii) by sequestering NFAT away from DNA (60) or by sequestering other NFAT partners, such as AP-1 (52) and (iii) by forming a FOXP3:NFAT cooperative complex (46)**.

FOXP3 can regulate gene expression of number of genes that are also targets for NFAT transcription factor. This activity was justified by the capacity of FOXP3 to interact physically with NFAT (60). The mechanism by which FOXP3 influences the expression of NFAT-dependent genes is under study. It has been suggested at least three modes of action of FOXP3 to inhibit the expression of genes regulated by NFAT: (i) by competing with NFAT for binding to DNA (77–79), (ii) by sequestering NFAT away from DNA (based on the finding that NFAT and FOXP3 physically interact in cell lysates (60)) or by sequestering other NFAT partners, such as AP-1 (52), and (iii) by forming a cooperative complex (46) (Figure 1). Regarding the third option, Wu and colleagues showed that the repressive effect of FOXP3 on cytokine gene expression and its activating effect on other genes, such as CTLA4 and CD25, is in fact driven by a cooperative complex formation between NFAT and FOXP3. NFAT binds cooperatively with transcription factors of the AP-1 (Fos/Jun) family to composite NFAT:AP-1 sites, found in the regulatory regions of many genes that are induced during lymphocyte activation (80). But, depending on its partner, NFAT seems to direct two entirely contrary biological programs: (i) T cell activation by recruiting AP-1 to the regulatory regions of appropriate target genes and (ii) T cell tolerance by recruiting FOXP3 (46). The cooperative complex FOXP3:NFAT is required to repress expression of the cytokine IL-2, upregulate expression of the Treg markers CTLA4 and CD25, and confer suppressor function (46). In a recent paper, Bandukwala et al. (55), solved the crystal structure of the FKH domain of FOXP3 bound to DNA in conjunction with the DNA-binding domain of its partner, NFAT1. This work showed that a region of FOXP3 identified as Wing1, and specially aminoacids 399–401 from FOXP3, inserts into the CX-E’F-fg groove of NFAT that contains a string of positively charged residues, K664, R665, K666, and R667 (55). Mutations that disrupt Foxp3 interaction with NFAT, interfere with the ability of FOXP3 to repress expression of the cytokine IL-2, upregulate expression of the Treg markers CTLA4 and CD25, and the suppressor function (46).

The FKH domain is also implicated in the nuclear localization of FOXP3. Indeed, it has been demonstrated that a C-terminal fragment of Foxp3 containing the entire FKH domain with short flanking sequences at each end was necessary and sufficient for import of FOXP3 to the nucleus (51). However, nuclear import of transcription factors are regulated at multiple levels, including post-translational modifications, binding and release from compartment-specific anchors, and the selective utilization of specific transport factors (81). It is not known whether FOXP3 nuclear translocation can be regulated by other transcription factors. Indeed, it has been shown that the interaction between Foxp3 and c-Rel may contribute to the nuclear translocation of FOXP3 (60, 61).

Forkhead domain of FOXP3 has been also implicated in the interaction with the hypoxia-inducible factor 1 (HIF-1α). It has been described that HIF-1 enhances Th17 development through direct transcriptional activation of RORγτ and via tertiary complex formation with RORγτ and p300 recruitment to the IL-17 promoter. HIF-1α attenuates T(reg) development by binding FOXP3 and targeting it for proteasomal degradation (42). These findings highlight the importance of metabolic cues in T cell fate determination and suggest that metabolic modulation could ameliorate certain T cell-based immune pathologies.

## FOXP3 Post-Translational Modifications

Post-translational modifications, including phosphorylation, acetylation, ubiquitination, sumoylation, methylation, and hydroxylation, are recognized as important determinants for the dynamic regulation of transcription factors (82). As mentioned above, FOXP3 activity can be regulated by acetylation, a process catalyzed by distinct types of HATs and non-histone proteins that are activated/inhibited in response to extrinsic cellular signals. It has been demonstrated that acetylation/deacetylation of FOXP3, in response to stimuli such as TGF-β or IL-6, affects FOXP3 stability and its promoter binding activity (83, 84). It is clear that HAT and HDACs, such as TIP60, p300, HDAC7, HDAC9, or SIRT1, are components of the Foxp3 interactome and are involved in regulating the acetylation status of Foxp3 [reviewed in (85)]. FOXP3 acetylation can increase its stability. Indeed, the histone acetyl transferase p300 competes for lysine residues with E3 ubiquitin ligase, which can target FOXP3 for its degradation (86). As mentioned above, FOXP3 degradation directed by HIF-1α during Th17 development (42).

FOXP3 activity can also be regulated by phosphorylation. The primary structure of FOXP3 contain four cyclin-dependent kinase (CDK) motifs (Ser/Thr-Pro) located within the N-terminal domain. It has been shown very recently that cyclin-dependent kinase 2 (CDK2) together with cyclin E can phophorylate these sites and regulate Foxp3 stability and affect negatively its suppressor function (87). It has also been demonstrated that phosphorylation of FOXP3 at serine 418 affects its transcriptional activity and Treg function (88). The authors showed in rheumatoid arthritis-derived Treg cells that Ser418 was specifically dephosphorylated by protein phosphatase 1 (PP1), whose expression and enzymatic activity ca be induced by tumor necrosis factor α (TNF-α) leading to impaired Treg-cell function. These data highlight how Treg-cell function may be subverted by pro-inflammatory cytokines such as TNF-α in inflammatory diseases, and how it can be reversed by TNF-α antagonism (88).

## FOXP3 as a Target for Drug Development

FOXP3 is a multifaceted transcription factor with a major role in the control of immune homeostasis mediated by Treg cells. But also, FOXP3 expression has been shown to be induced transitorily on CD4+ T cells upon TCR stimulation leading to hyporesponsiveness of the cell. This finding suggests that the induction of FOXP3 serves to shut off T cell activation. It is widely acknowledged that inhibiting Treg-cell function in patients with cancer is essential to improve the efficacy of anti-tumoral therapies. But, on the other hand, FOXP3 has emerged as an important regulator of some oncogenes and as a tumor suppressor factor able to control cell proliferation of tumor cells. Therefore, how should the function of Foxp3 be inhibited in Treg cells and effector T cells without altering the beneficial effects of Foxp3 on tumors?

**Figure 2:**
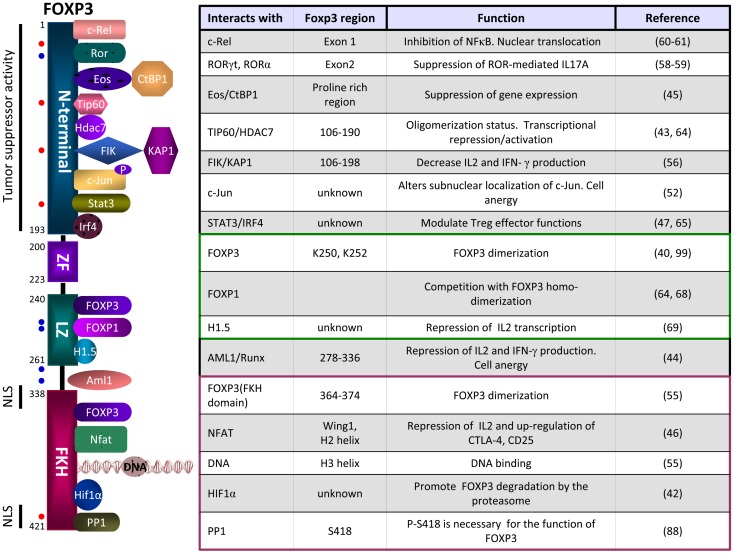
**Schematic representation of some of the FOXP3 partners, their site of interactions and functions**. The N-terminal domain of FOXP3 can interact with c-Rel (nuclear factor κB transcription factor c-Rel), RORγt (Retinoic acid receptor-related orphan receptor gamma t), RORα (Retinoid-related orphan receptor alpha), Eos (Ikaros family zinc finger 4, IKZF4), TIP60 (histone acetyltransferase TIP60), HDAC7 (histone deacetylase 7), FIK (FOXP3-interacting KRAB domain containing protein), c-Jun (transcription factor c-Jun), STAT3 (Signal transducer and activator of transcription 3), IRF4 (interferon regulatory factor 4). The Leucine zipper domain can interact with FOXP3, FOXP1, or H1.5 (Histone H1.5). The FKH domain can interact with FOXP3, NFAT (nuclear factor of activated T cells), the DNA or HIF-1α (Hypoxia-inducible factor 1α). Dots in red represent sites of phosphorylation and dots in blue represents sites of acetylation. NLS, nuclear localization sequences.

Several strategies have been proposed to neutralize Treg activity. Thus, it has been shown that administration of low doses of cyclophosphamide is able to deplete Treg and favors anti-tumor therapies (89–91). Also, targeting the alpha-subunit of the IL-2 receptor by using a fusion protein between the IL-2 and the diphtheria toxin has been tested in clinical trials with different results (89, 90). However, these strategies lack of high specificity and might eliminate both effector T cells and Treg cells. In addition, depletion of Treg cells by these strategies also raises the possibility of autoimmunity (91). Other approaches have been directed to inhibit factors produced by Tregs, such us transforming growth factor-β (TGF-β). We have shown that a peptide inhibitor of TGF-β inhibited Treg activity and improved protective anti-tumor immunogenicity elicited by a vaccine (92, 93). These data suggest that inhibition of TGF-β, in particular by small molecules that might penetrate the interface between contacting T cells, would be a valuable tool to inhibit Treg activity and consequently, these molecules might be useful to potentiate antiviral or anti-tumor immunotherapies. However, there are other options to regulate Treg activity by targeting FOXP3 directly. As it has been described above, FOXP3 orchestrate a transcriptional network in collaboration with a large number of cofactors, and thus, the complexity of FOXP3 interactome may determine the final outcome of the cell. In Figure 2 we summarize some of the cofactors identified as partners of FOXP3 and through which FOXP3 exerts some of its functions. Regulation of the members of the FOXP3 interactome may offer opportunities for the development of new treatments for autoimmune diseases, cancer, or infectious diseases. In a previous work, using a phage displayed peptide library we identified a 15-mer synthetic peptide, P60, able to enter into the cells, bind to FOXP3, inhibit FOXP3 nuclear translocation, and reduce its ability to suppress the transcription factors NF-κB and NFAT impairing Treg activity *in vitro* and *in vivo* (94). The specific region of FOXP3 which interacts with the peptide has not been elucidated yet, but it is clear that peptide P60 inhibits only certain number of functions ascribed to FOXP3, without altering others.

There are many opportunities to disrupt specific elements of FOXP3 interactome which should be investigated. Thus, small molecules targeting HAT/HDAC or disrupting their interactions with FOXP3 are interesting candidates for the regulation of Treg function in vaccines and tumor therapies (85). Similarly, inhibition of FOXP3 interaction with phospsphorylated c-Jun, Eos, c-Rel, FIK, ROR, AML1, or NFAT might lead to the inhibition of specific functions of FOXP3. Clearly, small molecules targeting these proteins are candidates for the regulation of Treg function in vaccines and tumor therapies.

Moreover, there are also opportunities to modulate the post-translational modifications of FOXP3 by regulating the activity of HAT/HDAC complex (95). Thus, several HDAC inhibitors have been proved to enhance the production and suppressive functions of FOXP3(+) regulatory T cells, being effective in murine models of arthritis, allograft rejection, and colitis (96–98). Similarly, modification of the phosphorylation status of FOXP3 may offer opportunities to modulate its activity. Strategies to enhance CDK2 and cyclin E activity might downregulate the stability and activity of FOXP3 and control Treg function (87). However, it must be emphasized that new drugs to inhibit FOXP3 functions should preserve its antiproliferative activities which are beneficial for the control of tumor cell growth.

## Conflict of Interest Statement

The authors declare that the research was conducted in the absence of any commercial or financial relationships that could be construed as a potential conflict of interest.

## References

[1] Kaufmann E Knochel W Five years on the wings of fork head. Mech Dev (1996) 57:3–20 10.1016/0925-4773(96)00539-4 8817449

[2] Clark KL Halay ED Lai E Burley SK Co-crystal structure of the HNF-3/fork head DNA-recognition motif resembles histone H5. Nature (1993) 364:412–20 10.1038/364412a0 8332212

[3] Kaestner KH Knochel W Martinez DE Unified nomenclature for the winged helix/forkhead transcription factors. Genes Dev (2000) 14:142–6 10.1101/gad.14.2.142 10702024

[4] Lehmann OJ Sowden JC Carlsson P Jordan T Bhattacharya SS Fox’s in development and disease. Trends Genet (2003) 19:339–44 10.1016/S0168-9525(03)00111-2 12801727

[5] Brunkow ME Jeffery EW Hjerrild KA Paeper B Clark LB Yasayko SA Disruption of a new forkhead/winged-helix protein, scurfin, results in the fatal lymphoproliferative disorder of the scurfy mouse. Nat Genet (2001) 27:68–73 10.1038/83784 11138001

[6] Bennett CL Christie J Ramsdell F Brunkow ME Ferguson PJ Whitesell L The immune dysregulation, polyendocrinopathy, enteropathy, X-linked syndrome (IPEX) is caused by mutations of FOXP3. Nat Genet (2001) 27:20–1 10.1038/83713 11137993

[7] Chatila TA Blaeser F Ho N Lederman HM Voulgaropoulos C Helms C JM2, encoding a fork head-related protein, is mutated in X-linked autoimmunity-allergic disregulation syndrome. J Clin Invest (2000) 106:R75–81 10.1172/JCI11679 11120765 PMC387260

[8] Wildin RS Ramsdell F Peake J Faravelli F Casanova JL Buist N X-linked neonatal diabetes mellitus, enteropathy and endocrinopathy syndrome is the human equivalent of mouse scurfy. Nat Genet (2001) 27:18–20 10.1038/83707 11137992

[9] Blair PJ Bultman SJ Haas JC Rouse BT Wilkinson JE Godfrey VL CD4+CD8− T cells are the effector cells in disease pathogenesis in the scurfy (sf) mouse. J Immunol (1994) 153:3764–74 7930593

[10] Sakaguchi S Sakaguchi N Asano M Itoh M Toda M Immunologic self-tolerance maintained by activated T cells expressing IL-2 receptor alpha-chains (CD25). Breakdown of a single mechanism of self-tolerance causes various autoimmune diseases. J Immunol (1995) 155:1151–64 7636184

[11] Suri-Payer E Amar AZ Thornton AM Shevach EM CD4+CD25+ T cells inhibit both the induction and effector function of autoreactive T cells and represent a unique lineage of immunoregulatory cells. J Immunol (1998) 160:1212–8 9570536

[12] Hori S Nomura T Sakaguchi S Control of regulatory T cell development by the transcription factor Foxp3. Science (2003) 299:1057–61 10.1126/science.1079490 12522256

[13] Khattri R Cox T Yasayko SA Ramsdell F An essential role for Scurfin in CD4+CD25+ T regulatory cells. Nat Immunol (2003) 4:337–42 10.1038/ni909 12612581

[14] Aandahl EM Michaelsson J Moretto WJ Hecht FM Nixon DF Human CD4+ CD25+ regulatory T cells control T-cell responses to human immunodeficiency virus and cytomegalovirus antigens. J Virol (2004) 78:2454–9 10.1128/JVI.78.5.2454-2459.2004 14963140 PMC369239

[15] Cabrera R Tu Z Xu Y Firpi RJ Rosen HR Liu C An immunomodulatory role for CD4(+)CD25(+) regulatory T lymphocytes in hepatitis C virus infection. Hepatology (2004) 40:1062–71 10.1002/hep.20454 15486925

[16] Curiel TJ Coukos G Zou L Alvarez X Cheng P Mottram P Specific recruitment of regulatory T cells in ovarian carcinoma fosters immune privilege and predicts reduced survival. Nat Med (2004) 10:942–9 10.1038/nm1093 15322536

[17] Liyanage UK Moore TT Joo HG Tanaka Y Herrmann V Doherty G Prevalence of regulatory T cells is increased in peripheral blood and tumor microenvironment of patients with pancreas or breast adenocarcinoma. J Immunol (2002) 169:2756–61 12193750 10.4049/jimmunol.169.5.2756

[18] Ormandy LA Hillemann T Wedemeyer H Manns MP Greten TF Korangy F Increased populations of regulatory T cells in peripheral blood of patients with hepatocellular carcinoma. Cancer Res (2005) 65:2457–64 10.1158/0008-5472.CAN-04-3232 15781662

[19] Viguier M Lemaitre F Verola O Cho MS Gorochov G Dubertret L Foxp3 expressing CD4+CD25(high) regulatory T cells are overrepresented in human metastatic melanoma lymph nodes and inhibit the function of infiltrating T cells. J Immunol (2004) 173:1444–53 15240741 10.4049/jimmunol.173.2.1444

[20] Woo EY Chu CS Goletz TJ Schlienger K Yeh H Coukos G Regulatory CD4(+)CD25(+) T cells in tumors from patients with early-stage non-small cell lung cancer and late-stage ovarian cancer. Cancer Res (2001) 61:4766–72 11406550

[21] Alvaro T Lejeune M Salvado MT Bosch R Garcia JF Jaen J Outcome in Hodgkin’s lymphoma can be predicted from the presence of accompanying cytotoxic and regulatory T cells. Clin Cancer Res (2005) 11:1467–73 10.1158/1078-0432.CCR-04-1869 15746048

[22] Badoual C Hans S Rodriguez J Peyrard S Klein C Agueznay Nel H Prognostic value of tumor-infiltrating CD4+ T-cell subpopulations in head and neck cancers. Clin Cancer Res (2006) 12:465–72 10.1158/1078-0432.CCR-05-1886 16428488

[23] Carreras J Lopez-Guillermo A Fox BC Colomo L Martinez A Roncador G High numbers of tumor-infiltrating FOXP3-positive regulatory T cells are associated with improved overall survival in follicular lymphoma. Blood (2006) 108:2957–64 10.1182/blood-2006-04-018218 16825494

[24] Ebert LM Tan BS Browning J Svobodova S Russell SE Kirkpatrick N The regulatory T cell-associated transcription factor FoxP3 is expressed by tumor cells. Cancer Res (2008) 68:3001–9 10.1158/0008-5472.CAN-07-5664 18413770

[25] Hinz S Pagerols-Raluy L Oberg HH Ammerpohl O Grussel S Sipos B Foxp3 expression in pancreatic carcinoma cells as a novel mechanism of immune evasion in cancer. Cancer Res (2007) 67:8344–50 10.1158/0008-5472.CAN-06-3304 17804750

[26] Niu J Jiang C Li C Liu L Li K Jian Z Foxp3 expression in melanoma cells as a possible mechanism of resistance to immune destruction. Cancer Immunol Immunother (2011) 60:1109–18 10.1007/s00262-011-1025-3 21547596 PMC11028752

[27] Zuo T Wang L Morrison C Chang X Zhang H Li W FOXP3 is an X-linked breast cancer suppressor gene and an important repressor of the HER-2/ErbB2 oncogene. Cell (2007) 129:1275–86 10.1016/j.cell.2007.04.034 17570480 PMC1974845

[28] Nair S Aldrich AJ McDonnell E Cheng Q Aggarwal A Patel P Immunologic targeting of FOXP3 in inflammatory breast cancer cells. PLoS One (2013) 8:e53150 10.1371/journal.pone.0053150 23341929 PMC3544902

[29] Wang L Liu R Li W Chen C Katoh H Chen GY Somatic single hits inactivate the X-linked tumor suppressor FOXP3 in the prostate. Cancer Cell (2009) 16:336–46 10.1016/j.ccr.2009.08.016 19800578 PMC2758294

[30] Zuo T Liu R Zhang H Chang X Liu Y Wang L FOXP3 is a novel transcriptional repressor for the breast cancer oncogene SKP2. J Clin Invest (2007) 117:3765–73 10.1172/JCI32538 18008005 PMC2075479

[31] Redpath M Xu B van Kempen LC Spatz A The dual role of the X-linked FoxP3 gene in human cancers. Mol Oncol (2011) 5:156–63 10.1016/j.molonc.2011.03.001 21489891 PMC5528280

[32] Karanikas V Speletas M Zamanakou M Kalala F Loules G Kerenidi T Foxp3 expression in human cancer cells. J Transl Med (2008) 6:19 10.1186/1479-5876-6-19 18430198 PMC2386447

[33] Gavin MA Rasmussen JP Fontenot JD Vasta V Manganiello VC Beavo JA Foxp3-dependent programme of regulatory T-cell differentiation. Nature (2007) 445:771–5 10.1038/nature05543 17220874

[34] Lin W Haribhai D Relland LM Truong N Carlson MR Williams CB Regulatory T cell development in the absence of functional Foxp3. Nat Immunol (2007) 8:359–68 10.1038/ni1445 17273171

[35] Marson A Kretschmer K Frampton GM Jacobsen ES Polansky JK MacIsaac KD Foxp3 occupancy and regulation of key target genes during T-cell stimulation. Nature (2007) 445:931–5 10.1038/nature05478 17237765 PMC3008159

[36] Zheng Y Josefowicz SZ Kas A Chu TT Gavin MA Rudensky AY Genome-wide analysis of Foxp3 target genes in developing and mature regulatory T cells. Nature (2007) 445:936–40 10.1038/nature05563 17237761

[37] Fu W Ergun A Lu T Hill JA Haxhinasto S Fassett MS A multiply redundant genetic switch ‘locks in’ the transcriptional signature of regulatory T cells. Nat Immunol (2012) 13:972–80 10.1038/ni.2420 22961053 PMC3698954

[38] Hill JA Feuerer M Tash K Haxhinasto S Perez J Melamed R Foxp3 transcription-factor-dependent and -independent regulation of the regulatory T cell transcriptional signature. Immunity (2007) 27:786–800 10.1016/j.immuni.2007.09.010 18024188

[39] Samstein RM Arvey A Josefowicz SZ Peng X Reynolds A Sandstrom R Foxp3 exploits a pre-existent enhancer landscape for regulatory T cell lineage specification. Cell (2012) 151:153–66 10.1016/j.cell.2012.06.053 23021222 PMC3493256

[40] Li B Samanta A Song X Iacono KT Brennan P Chatila TA FOXP3 is a homo-oligomer and a component of a supramolecular regulatory complex disabled in the human XLAAD/IPEX autoimmune disease. Int Immunol (2007) 19:825–35 10.1093/intimm/dxm043 17586580

[41] Rudra D deRoos P Chaudhry A Niec RE Arvey A Samstein RM Transcription factor Foxp3 and its protein partners form a complex regulatory network. Nat Immunol (2012) 13:1010–9 10.1038/ni.2402 22922362 PMC3448012

[42] Dang EV Barbi J Yang HY Jinasena D Yu H Zheng Y Control of T(H)17/T(reg) balance by hypoxia-inducible factor 1. Cell (2011) 146:772–84 10.1016/j.cell.2011.07.033 21871655 PMC3387678

[43] Li B Samanta A Song X Iacono KT Bembas K Tao R FOXP3 interactions with histone acetyltransferase and class II histone deacetylases are required for repression. Proc Natl Acad Sci U S A (2007) 104:4571–6 10.1073/pnas.0700298104 17360565 PMC1838642

[44] Ono M Yaguchi H Ohkura N Kitabayashi I Nagamura Y Nomura T Foxp3 controls regulatory T-cell function by interacting with AML1/Runx1. Nature (2007) 446:685–9 10.1038/nature05673 17377532

[45] Pan F Yu H Dang EV Barbi J Pan X Grosso JF Eos mediates Foxp3-dependent gene silencing in CD4+ regulatory T cells. Science (2009) 325:1142–6 10.1126/science.1176077 19696312 PMC2859703

[46] Wu Y Borde M Heissmeyer V Feuerer M Lapan AD Stroud JC FOXP3 controls regulatory T cell function through cooperation with NFAT. Cell (2006) 126:375–87 10.1016/j.cell.2006.05.042 16873067

[47] Zheng Y Chaudhry A Kas A deRoos P Kim JM Chu TT Regulatory T-cell suppressor program co-opts transcription factor IRF4 to control T(H)2 responses. Nature (2009) 458:351–6 10.1038/nature07674 19182775 PMC2864791

[48] Zhou Z Song X Li B Greene MI FOXP3 and its partners: structural and biochemical insights into the regulation of FOXP3 activity. Immunol Res (2008) 42:19–28 10.1007/s12026-008-8029-x 18626575

[49] Hori S The Foxp3 interactome: a network perspective of T(reg) cells. Nat Immunol (2012) 13:943–5 10.1038/ni.2424 22990900

[50] Gambineri E Torgerson TR Ochs HD Immune dysregulation, polyendocrinopathy, enteropathy, and X-linked inheritance (IPEX), a syndrome of systemic autoimmunity caused by mutations of FOXP3, a critical regulator of T-cell homeostasis. Curr Opin Rheumatol (2003) 15:430–5 10.1097/00002281-200307000-00010 12819471

[51] Lopes JE Torgerson TR Schubert LA Anover SD Ocheltree EL Ochs HD Analysis of FOXP3 reveals multiple domains required for its function as a transcriptional repressor. J Immunol (2006) 177:3133–42 16920951 10.4049/jimmunol.177.5.3133

[52] Lee SM Gao B Fang D FoxP3 maintains Treg unresponsiveness by selectively inhibiting the promoter DNA-binding activity of AP-1. Blood (2008) 111:3599–606 10.1182/blood-2007-09-115014 18223166

[53] Angel P Karin M The role of Jun, Fos and the AP-1 complex in cell-proliferation and transformation. Biochim Biophys Acta (1991) 1072:129–57 1751545 10.1016/0304-419x(91)90011-9

[54] Heinze E Chan G Mory R Khavari R Alavi A Chung SY Tumor suppressor and T-regulatory functions of Foxp3 are mediated through separate signaling pathways. Oncol Lett (2011) 2:665–8 10.3892/ol.2011.307 22848246 PMC3406440

[55] Bandukwala HS Wu Y Feuerer M Chen Y Barboza B Ghosh S Structure of a domain-swapped FOXP3 dimer on DNA and its function in regulatory T cells. Immunity (2011) 34:479–91 10.1016/j.immuni.2011.02.017 21458306 PMC3085397

[56] Huang C Martin S Pfleger C Du J Buckner JH Bluestone JA Cutting edge: a novel, human-specific interacting protein couples FOXP3 to a chromatin-remodeling complex that contains KAP1/TRIM28. J Immunol (2013) 190:4470–3 10.4049/jimmunol.1203561 23543754 PMC4197931

[57] Allan SE Passerini L Bacchetta R Crellin N Dai M Orban PC The role of 2 FOXP3 isoforms in the generation of human CD4+ Tregs. J Clin Invest (2005) 115:3276–84 10.1172/JCI24685 16211090 PMC1242190

[58] Ichiyama K Yoshida H Wakabayashi Y Chinen T Saeki K Nakaya M Foxp3 inhibits RORgammat-mediated IL-17A mRNA transcription through direct interaction with RORgammat. J Biol Chem (2008) 283:17003–8 10.1074/jbc.M801286200 18434325

[59] Zhou L Lopes JE Chong MM Ivanov II Min R Victora GD TGF-beta-induced Foxp3 inhibits T(H)17 cell differentiation by antagonizing RORgammat function. Nature (2008) 453:236–40 10.1038/nature06878 18368049 PMC2597437

[60] Bettelli E Dastrange M Oukka M Foxp3 interacts with nuclear factor of activated T cells and NF-kappa B to repress cytokine gene expression and effector functions of T helper cells. Proc Natl Acad Sci U S A (2005) 102:5138–43 10.1073/pnas.0501675102 15790681 PMC555574

[61] Loizou L Andersen KG Betz AG Foxp3 interacts with c-Rel to mediate NF-kappaB repression. PLoS One (2011) 6:e18670 10.1371/journal.pone.0018670 21490927 PMC3072406

[62] Fraser JD Irving BA Crabtree GR Weiss A Regulation of interleukin-2 gene enhancer activity by the T cell accessory molecule CD28. Science (1991) 251:313–6 10.1126/science.1846244 1846244

[63] Liou HC Hsia CY Distinctions between c-Rel and other NF-kappaB proteins in immunity and disease. Bioessays (2003) 25:767–80 10.1002/bies.10306 12879447

[64] Song X Li B Xiao Y Chen C Wang Q Liu Y Structural and biological features of FOXP3 dimerization relevant to regulatory T cell function. Cell Rep (2012) 1:665–75 10.1016/j.celrep.2012.04.012 22813742 PMC3401381

[65] Chaudhry A Rudra D Treuting P Samstein RM Liang Y Kas A CD4+ regulatory T cells control TH17 responses in a Stat3-dependent manner. Science (2009) 326:986–91 10.1126/science.1172702 19797626 PMC4408196

[66] Darce J Rudra D Li L Nishio J Cipolletta D Rudensky AY An N-terminal mutation of the Foxp3 transcription factor alleviates arthritis but exacerbates diabetes. Immunity (2012) 36:731–41 10.1016/j.immuni.2012.04.007 22579475 PMC3386606

[67] Chae WJ Henegariu O Lee SK Bothwell AL The mutant leucine-zipper domain impairs both dimerization and suppressive function of Foxp3 in T cells. Proc Natl Acad Sci U S A (2006) 103:9631–6 10.1073/pnas.0600225103 16769892 PMC1480458

[68] Wang B Lin D Li C Tucker P Multiple domains define the expression and regulatory properties of Foxp1 forkhead transcriptional repressors. J Biol Chem (2003) 278:24259–68 10.1074/jbc.M207174200 12692134

[69] Mackey-Cushman SL Gao J Holmes DA Nunoya JI Wang R Unutmaz D FoxP3 interacts with linker histone H1.5 to modulate gene expression and program Treg cell activity. Genes Immun (2011) 12:559–67 10.1038/gene.2011.31 21654845 PMC4329728

[70] Bennett CL Brunkow ME Ramsdell F O’Briant KC Zhu Q Fuleihan RL A rare polyadenylation signal mutation of the FOXP3 gene (AAUAAA →AAUGAA) leads to the IPEX syndrome. Immunogenetics (2001) 53:435–9 10.1007/s002510100358 11685453

[71] Harbuz R Lespinasse J Boulet S Francannet C Creveaux I Benkhelifa M Identification of new FOXP3 mutations and prenatal diagnosis of IPEX syndrome. Prenat Diagn (2010) 30:1072–8 10.1002/pd.2613 20842625

[72] Owen CJ Jennings CE Imrie H Lachaux A Bridges NA Cheetham TD Mutational analysis of the FOXP3 gene and evidence for genetic heterogeneity in the immunodysregulation, polyendocrinopathy, enteropathy syndrome. J Clin Endocrinol Metab (2003) 88:6034–9 10.1210/jc.2003-031080 14671208

[73] Rubio-Cabezas O Minton JA Caswell R Shield JP Deiss D Sumnik Z Clinical heterogeneity in patients with FOXP3 mutations presenting with permanent neonatal diabetes. Diabetes Care (2009) 32:111–6 10.2337/dc08-1188 18931102 PMC2606841

[74] Torgerson TR Linane A Moes N Anover S Mateo V Rieux-Laucat F Severe food allergy as a variant of IPEX syndrome caused by a deletion in a noncoding region of the FOXP3 gene. Gastroenterology (2007) 132:1705–17 10.1053/j.gastro.2007.02.044 17484868

[75] Bacchetta R Passerini L Gambineri E Dai M Allan SE Perroni L Defective regulatory and effector T cell functions in patients with FOXP3 mutations. J Clin Invest (2006) 116:1713–22 10.1172/JCI25112 16741580 PMC1472239

[76] Koh KP Sundrud MS Rao A Domain requirements and sequence specificity of DNA binding for the forkhead transcription factor FOXP3. PLoS One (2009) 4:e8109 10.1371/journal.pone.0008109 19956618 PMC2779587

[77] Coffer PJ Burgering BM Forkhead-box transcription factors and their role in the immune system. Nat Rev Immunol (2004) 4:889–99 10.1038/nri1488 15516968

[78] Schubert LA Jeffery E Zhang Y Ramsdell F Ziegler SF Scurfin (FOXP3) acts as a repressor of transcription and regulates T cell activation. J Biol Chem (2001) 276:37672–9 10.1074/jbc.M104521200 11483607

[79] Torgerson TR Genin A Chen C Zhang M Zhou B Anover-Sombke S FOXP3 inhibits activation-induced NFAT2 expression in T cells thereby limiting effector cytokine expression. J Immunol (2009) 183:907–15 10.4049/jimmunol.0800216 19564342 PMC2778477

[80] Rao A Luo C Hogan PG Transcription factors of the NFAT family: regulation and function. Annu Rev Immunol (1997) 15:707–47 10.1146/annurev.immunol.15.1.707 9143705

[81] Lee SH Hannink M Molecular mechanisms that regulate transcription factor localization suggest new targets for drug development. Adv Drug Deliv Rev (2003) 55:717–31 10.1016/S0169-409X(03)00052-8 12788536

[82] Huang B Yang XD Lamb A Chen LF Posttranslational modifications of NF-kappaB: another layer of regulation for NF-kappaB signaling pathway. Cell Signal (2010) 22:1282–90 10.1016/j.cellsig.2010.03.017 20363318 PMC2893268

[83] Samanta A Li B Song X Bembas K Zhang G Katsumata M TGF-beta and IL-6 signals modulate chromatin binding and promoter occupancy by acetylated FOXP3. Proc Natl Acad Sci U S A (2008) 105:14023–7 10.1073/pnas.0806726105 18779564 PMC2544572

[84] van Loosdregt J Vercoulen Y Guichelaar T Gent YY Beekman JM van Beekum O Regulation of Treg functionality by acetylation-mediated Foxp3 protein stabilization. Blood (2010) 115:965–74 10.1182/blood-2009-02-207118 19996091

[85] Xiao Y Li B Zhou Z Hancock WW Zhang H Greene MI Histone acetyltransferase mediated regulation of FOXP3 acetylation and Treg function. Curr Opin Immunol (2010) 22:583–91 10.1016/j.coi.2010.08.013 20869864 PMC2967626

[86] Kwon HS Lim HW Wu J Schnolzer M Verdin E Ott M Three novel acetylation sites in the Foxp3 transcription factor regulate the suppressive activity of regulatory T cells. J Immunol (2012) 188:2712–21 10.4049/jimmunol.1100903 22312127 PMC3478122

[87] Morawski PA Mehra P Chen C Bhatti T Wells AD Foxp3 Protein Stability Is Regulated by Cyclin-dependent Kinase 2. J Biol Chem (2013) 288:24494–502 10.1074/jbc.M113.467704 23853094 PMC3750148

[88] Nie H Zheng Y Li R Guo TB He D Fang L Phosphorylation of FOXP3 controls regulatory T cell function and is inhibited by TNF-alpha in rheumatoid arthritis. Nat Med (2013) 19:322–8 10.1038/nm.3085 23396208

[89] Attia P Maker AV Haworth LR Rogers-Freezer L Rosenberg SA Inability of a fusion protein of IL-2 and diphtheria toxin (Denileukin Diftitox, DAB389IL-2, ONTAK) to eliminate regulatory T lymphocytes in patients with melanoma. J Immunother (2005) 28:582–92 10.1097/01.cji.0000175468.19742.10 16224276 PMC1533764

[90] Dannull J Su Z Rizzieri D Yang BK Coleman D Yancey D Enhancement of vaccine-mediated antitumor immunity in cancer patients after depletion of regulatory T cells. J Clin Invest (2005) 115:3623–33 10.1172/JCI25947 16308572 PMC1288834

[91] Ruddle JB Harper CA Honemann D Seymour JF Prince HM A denileukin diftitox (Ontak) associated retinopathy? Br J Ophthalmol (2006) 90:1070–1 10.1136/bjo.2006.091165 16854841 PMC1857180

[92] Gil-Guerrero L Dotor J Huibregtse IL Casares N Lopez-Vazquez AB Rudilla F In vitro and in vivo down-regulation of regulatory T cell activity with a peptide inhibitor of TGF-beta1. J Immunol (2008) 181:126–35 18566377 10.4049/jimmunol.181.1.126

[93] Llopiz D Dotor J Casares N Bezunartea J Diaz-Valdes N Ruiz M Peptide inhibitors of transforming growth factor-beta enhance the efficacy of antitumor immunotherapy. Int J Cancer (2009) 125:2614–23 10.1002/ijc.24656 19530254

[94] Casares N Rudilla F Arribillaga L Llopiz D Riezu-Boj JI Lozano T A peptide inhibitor of FOXP3 impairs regulatory T cell activity and improves vaccine efficacy in mice. J Immunol (2010) 185:5150–9 10.4049/jimmunol.1001114 20870946

[95] Tao R de Zoeten EF Ozkaynak E Chen C Wang L Porrett PM Deacetylase inhibition promotes the generation and function of regulatory T cells. Nat Med (2007) 13:1299–307 10.1038/nm1652 17922010

[96] de Zoeten EF Wang L Sai H Dillmann WH Hancock WW Inhibition of HDAC9 increases T regulatory cell function and prevents colitis in mice. Gastroenterology (2010) 138:583–94 10.1053/j.gastro.2009.10.037 19879272 PMC3369426

[97] Saouaf SJ Li B Zhang G Shen Y Furuuchi N Hancock WW Deacetylase inhibition increases regulatory T cell function and decreases incidence and severity of collagen-induced arthritis. Exp Mol Pathol (2009) 87:99–104 10.1016/j.yexmp.2009.06.003 19577564 PMC2753738

[98] Wang L Tao R Hancock WW Using histone deacetylase inhibitors to enhance Foxp3(+) regulatory T-cell function and induce allograft tolerance. Immunol Cell Biol (2009) 87:195–202 10.1038/icb.2008.106 19172156

